# Orally Delivered Scorpion Antimicrobial Peptides Exhibit Activity against Pea Aphid (*Acyrthosiphon pisum*) and Its Bacterial Symbionts

**DOI:** 10.3390/toxins9090261

**Published:** 2017-08-24

**Authors:** Karen Luna-Ramirez, Marisa Skaljac, Jens Grotmann, Phillipp Kirfel, Andreas Vilcinskas

**Affiliations:** 1 Fraunhofer Institute for Molecular Biology and Applied Ecology (IME), Bioresources Project Group, Winchesterstrasse 2, 35394 Giessen, Germany; ramirezk@uow.edu.au (K.L.-R.); marisa.skaljac@ime.fraunhofer.de (M.S.); jens.grotmann@ime.fraunhofer.de (J.G.); phillipp.kirfel@ime.fraunhofer.de (P.K.); 2 Institute for Insect Biotechnology, Justus Liebig University of Giessen, Heinrich-Buff-Ring 26-32, 35392 Giessen, Germany

**Keywords:** *Acyrthosiphon pisum*, scorpion toxins, symbiosis, antimicrobial peptides

## Abstract

Aphids are severe agricultural pests that damage crops by feeding on phloem sap and vectoring plant pathogens. Chemical insecticides provide an important aphid control strategy, but alternative and sustainable control measures are required to avoid rapidly emerging resistance, environmental contamination, and the risk to humans and beneficial organisms. Aphids are dependent on bacterial symbionts, which enable them to survive on phloem sap lacking essential nutrients, as well as conferring environmental stress tolerance and resistance to parasites. The evolution of aphids has been accompanied by the loss of many immunity-related genes, such as those encoding antibacterial peptides, which are prevalent in other insects, probably because any harm to the bacterial symbionts would inevitably affect the aphids themselves. This suggests that antimicrobial peptides (AMPs) could replace or at least complement conventional insecticides for aphid control. We fed the pea aphids (*Acyrthosiphon pisum*) with AMPs from the venom glands of scorpions. The AMPs reduced aphid survival, delayed their reproduction, displayed in vitro activity against aphid bacterial symbionts, and reduced the number of symbionts in vivo. Remarkably, we found that some of the scorpion AMPs compromised the aphid bacteriome, a specialized organ that harbours bacterial symbionts. Our data suggest that scorpion AMPs holds the potential to be developed as bio-insecticides, and are promising candidates for the engineering of aphid-resistant crops.

## 1. Introduction

Aphids are among the most destructive agricultural pests, causing direct damage to crops by feeding on phloem, as well as indirect losses by transmitting viruses [1]. Aphids are also biological models for the investigation of insect–plant interactions and symbiosis [2]. *Buchnera aphidicola* is an obligate bacterial symbiont of aphids, and is exclusively localized in a specialized structure known as bacteriome, which consists of bacteriocytes. This species has coevolved with aphids to provide them with essential amino acids that are not supplied in sufficient quantities by the sugar-rich phloem sap on which aphids feed [3,4]. Aphids also frequently host one or more secondary bacterial symbionts, including *Serratia symbiotica*, *Hamiltonella defensa*, and *Regiella insecticola* [4]. These symbionts colonize different aphid tissues and provide several functions, including protection against natural enemies, heat stress tolerance, a supply of nutrients, and adaptation to the host plant [5,6,7].

The pea aphid *Acyrthosiphon pisum* (Harris) was selected among more than 4700 known aphid species for the first aphid genome sequencing project [8]. The most surprising revelation was that *A. pisum* has a greatly reduced repertoire of innate immunity genes when compared to other insects [9,10,11]. The *A. pisum* genome lacks genes encoding classical antimicrobial peptides (AMPs), and also lacks components of the immune deficiency pathway [10]. However, *A. pisum* is not completely defenceless against pathogens because it has several genes encoding thaumatins, which confer antifungal activity in other insects [9]. Recently, genes encoding short peptides resembling AMPs were identified in the aphid bacteriome, and these may be used to control bacterial symbionts [12]. Nevertheless, the limited innate immune system in *A. pisum* is likely to represent a protective adaptation that helps to maintain long-lasting symbiosis with bacteria [10,11,13,14]. Accordingly, AMPs may provide the basis for alternative insecticides that can be expressed in crops, given that any harm to symbionts would inevitably affect the aphids themselves [15,16,17]. This strategy relies on the ability of orally ingested AMPs to function correctly, even when exposed to peptidases and proteases found in the aphid gut [18].

Scorpions are predatory arachnids that feed on small arthropods (mainly insects). Their venom components have evolved over more than 450 million years into specialized toxins that efficiently kill their prey [19]. Their venom contains a cocktail of bioactive compounds, including neurotoxins that target mammals and/or insects, and amongst others, AMPs. Scorpion AMPs belong to the non-disulfide-bridged peptides (NDBP) family, which have diverse biological functions, including antimicrobial, bradykinin-potentiating, and immunomodulatory activities [20,21,22,23]. Scorpion AMPs have only been investigated against human pathogenic bacteria showing low minimal inhibitory concentrations (MICs), even against multi-drug-resistant bacteria [20,21]. However, their use as antibiotics has proven challenging due to their mildly haemolytic activity [19]. 

Given that scorpions prey on insects, we attempted to broaden the use of scorpion AMPs by investigating their insecticidal activity against *A. pisum.* Several AMPs recently identified in the venom gland transcriptome of the scorpion *Urodacus yaschenkoi* (Birula) (UyCT1, UyCT3, UyCT5, Uy17, Uy192 and Uy234) have been produced as synthetic peptides and tested in vitro against human pathogenic bacteria [24,25,26]. Another AMP (Um4) was identified in the venom of the black rock scorpion *Urodacus manicatus* (Thorell) [27]. The antimicrobial activities of these naturally occurring scorpion AMPs were compared to modified analogues (designed peptides, herein named D-peptides) generated by exchanging some amino acids and inserting positively charged residues to increase the net positive charge of the AMPs, and hence their affinity for bacterial membranes. None of the natural or engineered scorpion AMPs were active against fungi, but many were active at low MICs (0.25–30 µM) against seven different bacteria [28].

Here, we selected scorpion AMPs with low MICs and low haemolytic activity in order to test their activity against aphids and aphid bacterial symbionts both in vitro and in vivo. Their activities were compared to three insect-derived AMPs, as well as the antibiotic rifampicin and the insecticide imidacloprid. Our data suggest that scorpion-derived AMPs are promising candidates for the development of bio-insecticides and aphid-resistant transgenic plants.

## 2. Results

### 2.1. Effect of AMP Treatments on Aphid Survival

**Figure 1:**
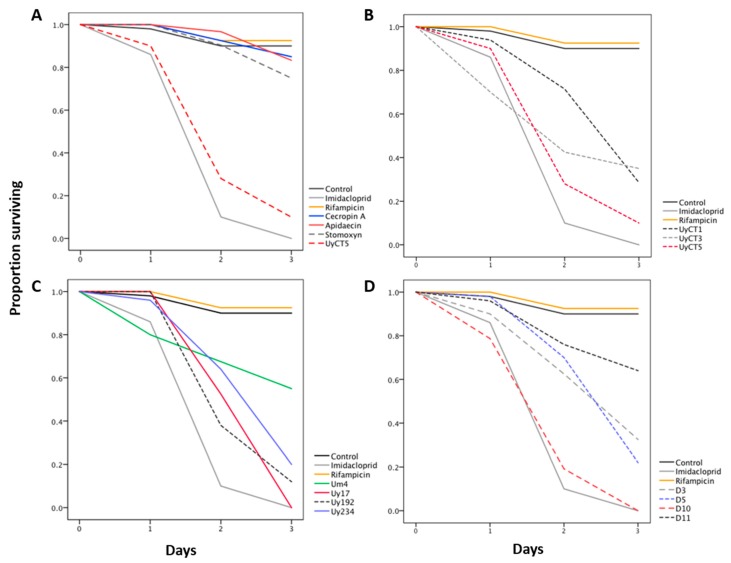
Insecticidal activity of scorpion and insect AMPs in *A. pisum*. Aphid survival was monitored during three days of feeding on an AP3 diet mixed with the corresponding AMP. Survival data were evaluated by Kaplan-Meier analysis. Statistical data are shown in Table S1. The insecticide imidacloprid was used as a positive control (5 µg/mL). (**A**) Insect AMPs, 500 µg/mL. (**B**,**C**) Natural scorpion AMPs, 500 µg/mL. (**D**) Designed scorpion AMPs (D-peptides), 500 µg/mL. The most effective AMPs were UyCT5, Uy17, Uy192, and D10, causing ~90% mortality. Insect AMPs had no significant effect on aphid survival.

The effect of each AMP was determined by tracking aphid survival during three days of feeding (Figure 1, Table 1 and Table S1). The insect AMPs did not affect aphid survival, whereas the scorpion AMPs were highly effective in killing the aphids. Some of the scorpion AMP treatments (UyCT3, UyCT5, and D3) were highly effective at all tested concentrations, whereas others (Uy17, Uy192, Uy234, D5, D10, and D11) were effective only at the medium (250 µg/mL) and high (500 µg/mL) concentrations, and UyCT1 and Um4 were only effective at the highest concentration.

We used the insecticide imidacloprid and the antibiotic rifampicin as controls to gauge the effectiveness of AMP treatments, although their modes of action differ from AMPs. Imidacloprid killed all the aphids in less than three days (survival rate 0%), whereas rifampicin did not significantly affect aphid survival as compared to the control AP3 diet (survival rate 92.5%).

Survival curves were constructed to compare the insecticidal activity of scorpion and insect AMPs in *A. pisum* (Figure 1, Table S1). Figure 1A shows the effect of the insect AMPs (500 µg/mL) compared with the control AP3 diet, imidacloprid (5 µg/mL) and scorpion AMP UyCT5 (500 µg/mL). The three insect AMPs (cecropin A, apidaecin and stomoxyn) had no significant effect (survival rate ≥75%) against *A. pisum* whereas UyCT5 was highly effective at the same concentration (survival rate <10%). Figure 1B compares three UyCT AMPs, which reduced aphid survival by 65–90%. UyCT5 was the most effective, killing 10% of the nymphs after the first day and more on the second and third days until only ~10% of the aphids survived. Figure 1C compares the *U. yaschenkoi* AMPs Uy17, Uy192, Uy234 and the *U. manicatus* AMP Um4, revealing that Uy17 was the most potent. Figure 1D compares the D-peptides, indicating that D10 was the most effective, killing all aphids by the end of the third day.

These data show that some of the scorpion AMPs are comparable to imidacloprid in terms of potency, e.g., Uy17 and Uy234 at the highest concentration, and D10 at the medium and highest concentrations, resulting in 100% mortality (Figure 1). Aphids that survived the three days of treatment were monitored for the following two weeks in order to detect any delayed effects of the AMP treatments. In most cases, there was no significant difference in survival as compared to the control AP3 diet. However, the survival rate continued to decline in the aphid groups fed on 50 μg/mL Um4, 50 and 500 μg/mL Uy234, and 500 μg/mL D5 (data not shown).

### 2.2. Effect of AMP Treatments on Aphid Reproduction

**Figure 2:**
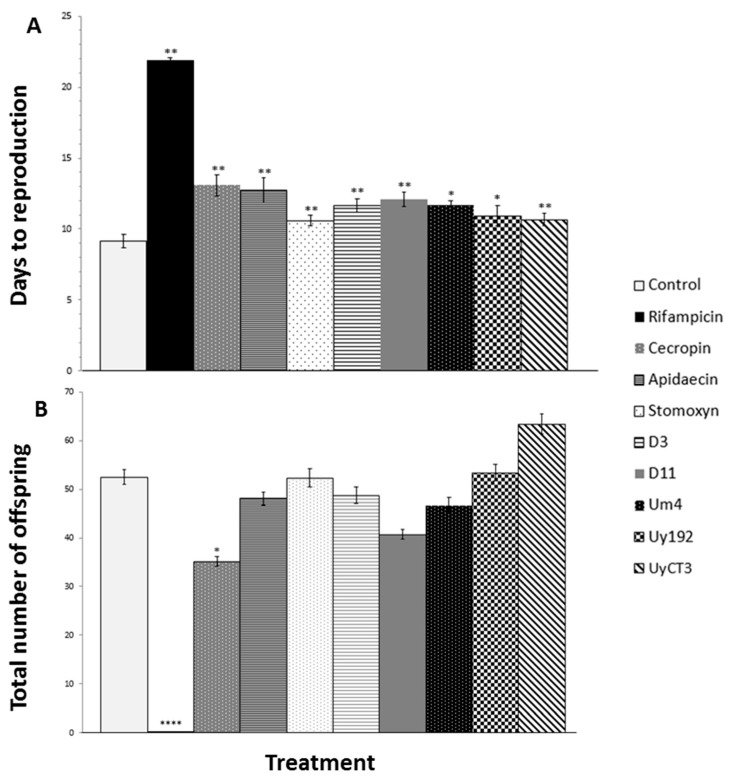
Impact of representative AMPs and antibiotic treatments on the time to reproduction and the number of offspring in *A. pisum*. (**A**) Days to reproduction increased after the treatments. (**B**) The number of offspring was not significantly affected by most of the treatments, except rifampicin and cecropin A. Negative control = control (AP3 diet); positive control = 50 µg/mL rifampicin; AMP treatments = 250 µg/mL. Statistical significance: * *p* < 0.05, ** *p* < 0.01, **** *p* < 0.0001.

The effect of each AMP on *A. pisum* reproduction was determined by counting the number of offspring and recording the delay before reproduction in surviving aphids for two weeks after treatment (Figure 2).

Most of the scorpion and insect AMP treatments affected the time to reproduction, in some cases even at the lowest tested concentration of 50 µg/mL, resulting in significant delays of several days as compared to the control AP3 diet (Figure 2A). The treatments that did not cause a significant reproductive delay were 500 µg/mL UyCT1, 500 µg/mL UyCT5, 500 µg/mL Uy192, 50 µg/mL Uy234, 500 µg/mL D3, and 500 µg/mL D5. 

Most of the treatments did not affect the number of offspring regardless of the effect on survival (Figure 2B). However, all three concentrations of cecropin A, the 500 µg/mL apidaecin treatment, and the 500 µg/mL Uy234 treatment had a significant impact on the number of offspring.

Rifampicin caused the most significant effect on reproduction, resulting in smaller and underdeveloped adults that produced hardly any offspring (Figure 2B). However, rifampicin did not affect aphid survival after three days of feeding nor during the two weeks after treatment (data not shown).

### 2.3. Effect of AMPs on Bacterial Growth In Vitro 

The susceptibility of aphid symbionts to AMPs was tested using the only known cultivable strain for aphids: *S. symbiotica* CWBI-2.3 [29]. Most of the scorpion AMPs that affected aphids in the feeding experiments also showed in vitro activity against *S. symbiotica* CWBI-2.3, with MICs of 125–500 μg/mL corresponding to the in vivo range (Table 2). Interestingly, some scorpion AMPs that were active in the feeding assays showed no in vitro activity against *S. symbiotica* CWBI-2.3, even at the highest tested concentration of 500 μg/mL (Uy234, Um4, D10 and D11) suggesting that they affect the aphids without targeting these bacterial symbionts. Further research is required to determine the mode of action of such compounds. Insect AMPs were not active against *S. symbiotica* CWBI-2.3, even at 500 μg/mL.

### 2.4. qPCR-Based Quantification of Bacterial Symbionts in Treated Aphids

**Figure 3:**
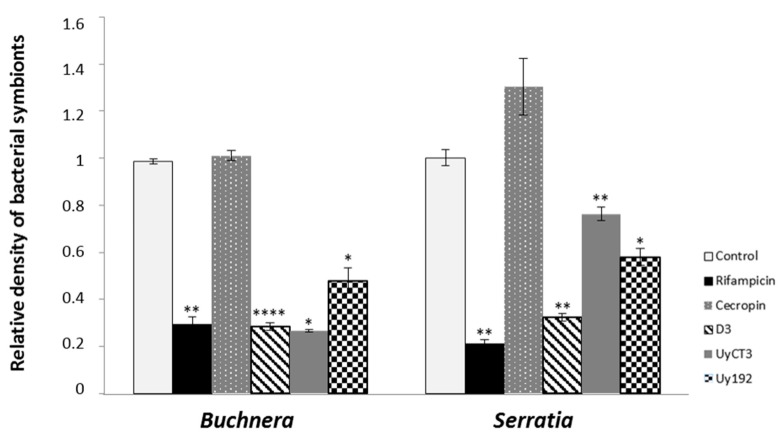
Quantitative PCR for the detection of *Buchnera aphidicola* and *Serratia symbiotica* in the *A. pisum* after AMP and antibiotic treatments. Data show the relative abundance of symbionts after three days of exposure for representative treatments (left panel = *B. aphidicola*; right panel = *S. symbiotica*). Negative control = control AP3 diet; positive control = 50 µg/mL rifampicin; AMP treatments = 250 µg/mL. Statistical significance indicated as follows: * *p* < 0.05, ** *p* < 0.01, **** *p* < 0.0001.

We used a quantitative PCR (qPCR) assay to determine the impact of scorpion AMPs on the population density of *S. symbiotica* and *B. aphidicola* in vivo. Two groups of samples were analysed: (i) after three days of AMP treatments in feeding chambers, and (ii) two weeks after treatment. These samples were compared to investigate whether *S. symbiotica* and *B. aphidicola* can recover from AMP exposure. As shown in Figure 3, many scorpion AMPs significantly reduced the density of *S. symbiotica* and *B. aphidicola* after three days as compared to the control AP3 diet: 50, and 250 µg/mL D3, 50, and 500 µg/mL D11, all three tested concentrations of UyCT3, and UyCT5, 250 µg/mL Uy192, and 500 µg/mL Um4. Furthermore, the density of symbionts was still significantly lower than the control level after two weeks in the groups treated with 50 µg/mL D3, 250, and 500 µg/mL D11, 250, and 500 µg/mL Um4, and all three concentrations of UyCT3 and UyCT5 (data not shown). 

Rifampicin caused a significant reduction in the numbers of *S. symbiotica* and *B. aphidicola* after three days of exposure and two weeks after treatment, but none of the insect AMPs reduced the density of either symbiont. Indeed, both symbionts were slightly more abundant two weeks after the treatment with cecropin A.

### 2.5. Localization of Bacterial Symbionts in A. pisum by Fluorescence In Situ Hybridization

**Figure 4:**
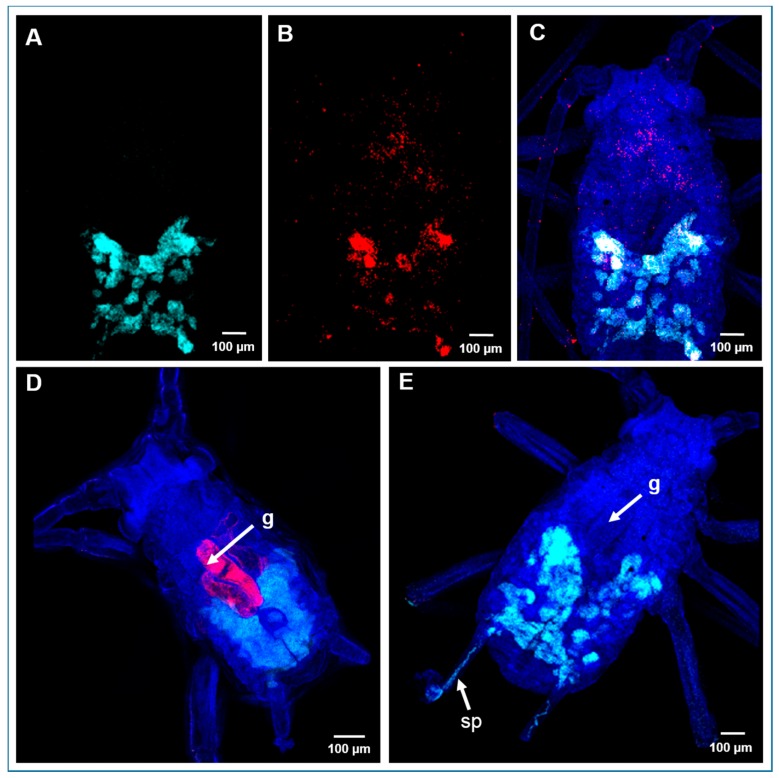
Localization of bacterial symbionts by fluorescence in situ hybridization (FISH) in *A. pisum* nymphs before and after treatment with scorpion AMPs. Specific probes were used for *Buchnera aphidicola* (light blue) and *Serratia symbiotica* (red). (**A**) Detection of *B. aphidicola*. (**B**) Detection of *S. symbiotica*. (**C**) Double FISH for the detection of both symbionts in untreated (control AP3 diet) *A. pisum* nymphs. (**D**) *S. symbiotica* and *B. aphidicola* in aphid nymphs after exposure to scorpion AMPs (e.g., UyCT3) for 24 h, and (**E**) 48 h. DAPI (dark blue) was used as a nuclear counterstain. Abbreviations: g = gut; sp = siphunculi.

Fluorescence in situ hybridization (FISH) was carried out with specific probes (Table S2) to establish the tissue distribution of *S. symbiotica* and *B. aphidicola* in aphids 24 and 48 h after exposure to the highest concentration of each AMP and the control treatments. In the negative control (AP3 diet) group, we found that *B. aphidicola* was exclusively localized in bacteriome of the nymphs, and its associated ovarioles (Figure 4A,C), whereas *S. symbiotica* was detected in most tissues, including the gut, bacteriome, and ovarioles (Figure 4B,C). The *S. symbiotica* signal remained visible in aphid tissues 24 h after the AMP treatments, and was prevalent in the gut (Figure 4D). However, the signal could not be detected after 48 h, indicating that the *S. symbiotica* 16*S* rRNA had degraded by this point (Figure 4E). We also found that treating aphids with the scorpion AMPs compromised the structure of the bacteriome (Figure 4E). Rifampicin treatment also reduced the *S. symbiotica* signal after 48 h, whereas the signal remained strong 48 h after treatment with the three insect AMPs (data not shown). 

In contrast to the results observed for *S. symbiotica*, neither the AMPs nor rifampicin reduced the intensity of the signal for *B. aphidicola* in aphid nymphs. However, the *B. aphidicola* signal was often detected in the siphunculi 48 h after treatment with D3, D5, D10, Um4, UyCT3, and UyCT5 (Figure 4E), which might indicate that the bacteriome structure has been compromised. As expected, imidacloprid did not affect the localization of the bacterial symbionts, because it acts directly on the insect central nervous system.

## 3. Discussion

Aphids are dependent on their association with bacterial symbionts, and antibiotics can therefore impair their fitness and fecundity [16,30]. The evolution of innate immunity in aphids has been accompanied by the loss of many genes encoding antibacterial peptides because their expression could damage bacterial symbionts [14]. This has led to a hypothesis in which engineered crops expressing AMPs could be used to target aphids via their bacterial symbionts [31,32]. Engineered pathogen-resistant crops already provide a sustainable strategy to counteract specific plant diseases. For example, the antifungal peptides gallerimycin from *Galleria mellonella* (Linnaeus) and metchnikowin from *Drosophila melanogaster* (Meigen) have been shown to confer fungal resistance in plants [33,34].

As previously stated, the efficacy of AMPs expressed in crops relies on the ability of orally ingested AMPs to function correctly even following exposure to digestive enzymes found in the aphid gut [18]. We therefore investigated whether feeding aphids with scorpion and insect AMPs can affect their survival and fecundity. We selected AMPs from two Australian scorpion species (*U. yaschenkoi* and *U. manicatus*) because the evolution of scorpions has involved the development of venom glands producing peptides and proteins that can efficiently kill insect prey [24,26]. Certain scorpion AMPs are also active against human pathogenic bacteria [21,28]. We used three insect AMPs (cecropin A, apidaecin and stomoxyn), as well as a synthetic insecticide (imidacloprid) and antibiotic (rifampicin) as controls to evaluate the scorpion AMPs.

Each of the scorpion AMPs we tested was active against *A. pisum*, affecting their survival and/or fecundity. UyCT3, UyCT5, and D3 were highly effective at all three tested concentrations, whereas UyCT1 and Um4 were effective only at the highest concentration (500 µg/mL). In contrast, the insect AMPs we tested had no effect on aphid survival, and only a minimal impact on reproduction (Figure 2). In addition, many of the tested scorpion and insect AMPs delayed reproduction, but only a few reduced the number of offspring (cecropin A, apidaecin and Uy234) (Figure 2). The impact of scorpion and insect AMPs on aphid reproduction is probably a non-specific consequence of AMP toxicity, which causes an overall decrease in the fitness of aphids, and thus impairs their reproductive ability.

One potential explanation for the differential activity of scorpion and insect AMPs against aphids and their bacterial symbionts is the origin and intrinsic characteristics of these peptides. Scorpion AMPs are short cationic amphipathic peptides that are produced in the venom gland [23]. They target cell membrane by a pore-forming mechanism resulting in the loss of electrolytes [35]. Their broad activity against bacteria, erythrocytes, and other mammalian cells has been attributed to their lack of selectivity. Their precise function in nature still remains unclear, but they may protect the telson (open end of the fifth metasomal segment) from bacterial infections and may also help neurotoxins reach their targets once the AMP has ruptured the cell membrane [19,36].

The insect AMPs used in this study are expressed in the haemolymph when the host insect is challenged by a pathogen [37,38,39,40]. These AMPs act selectively against the membranes of a wide range of human, animal, and plant bacterial pathogens, but they do not affect eukaryotic cells [41,42,43]. Insect AMPs usually disrupt bacterial membranes by forming pores, but the mechanism of apidaecin is different [35]. This proline-rich AMP not only breaches the bacterial membrane, but also binds intracellular targets. The ineffectiveness of insect AMPs in aphids may reflect their selective nature toward pathogens, whereas scorpion AMPs target different tissues, including the bacteriome, probably using the same lytic mode of action.

As well as assessing the impact of each AMP on aphid survival and fecundity, we evaluated their direct effect against both *B. aphidicola* and *S. symbiotica*. The CWBI-2.3 strain of *S. symbiotica* is the only aphid symbiont that can be cultivated under laboratory conditions [29]. This strain is a transitional form between a free-living bacterium and a host-dependent mutualistic symbiont, and is a close relative of the *S. symbiotica* strain found in the *A. pisum* population used in this study [44]. We were able to determine MICs for each AMP against *S. symbiotica* CWBI-2.3 in vitro. Most of the scorpion AMPs (UyCT1, UyCT3, UyCT5, Uy17, Uy192, D3, and D5) inhibited the growth of *S. symbiotica* CWBI-2.3 (Table 2). We found that several of the scorpion AMPs that affected aphid performance in the feeding assays were also active against *S. symbiotica* in vitro and in vivo, whereas others (Uy234, Um4, D10, and D11) did not act directly against the symbiont but were nevertheless active against the aphids in feeding assays, suggesting an alternative mechanism of action or an alternative target.

We also investigated the effect of the AMPs by using qPCR and FISH to directly characterize the population density and localization of both *B. aphidicola* and *S. symbiotica* in aphid tissues. FISH analysis did not reveal any clear AMP-mediated effect on the abundance of intracellular *B. aphidicola*, but there was a remarkable reduction in the *S. symbiotica* population, which was more accessible to the AMPs due to its intracellular and extracellular localization (Figure 4D,E). However, qPCR revealed a significant reduction in the density of both populations, confirming the antibacterial effect of the tested scorpion AMPs (Figure 3). 

The compartmentalization of symbionts inside the bacteriome and specialized host-derived membranes is an evolutionary strategy to protect mutualistic symbionts from host innate immunity, including AMPs [45]. This special structure must be breached before AMPs can exert their antibacterial activity [46,47,48,49]. For these reasons, the selective insect AMPs were probably unable to reach the bacterial symbionts, whereas the non-selective scorpion AMPs were more likely to compromise the bacteriome, affecting both symbionts (Figure 3 and Figure 4E). 

The observed insecticidal and antibacterial activities of scorpion AMPs against *A. pisum* and its bacterial symbionts are supported by earlier research in which indolicidin, an AMP from bovine neutrophils, showed activity against the green peach aphid *Myzus persicae* (Sulzer) and also affected the bacteriome [50]. Furthermore, scorpion AMPs (UyCT3, UyCT5, Uy192, Um4, D11) and indolicin showed activity against *Escherichia coli*, which is closely related to *B. aphidicola*, providing further support for our observations [20,21,28,51,52]. 

In summary, we found that the scorpion AMPs UyCT3, UyCT5, and D3 were the most effective against aphids and their symbionts. These AMPs showed insecticidal activity at different concentrations and they clearly affected aphid survival and reproduction, but also significantly reduced the population size of both *B. aphidicola* and *S. symbiotica*. There is a growing interest in the development of bio-insecticides derived from the venom of arachnids that prey on insects [53,54,55,56]. The natural characteristics of scorpion AMPs make them attractive candidates for this purpose because they are short and linear, and therefore easy to synthesize at low costs. Scorpion AMPs are also suitable candidates for the engineering of aphid-resistant crops, although further research is required to determine whether there are any negative effects in the plants themselves and whether the scorpion AMPs confer a significant degree of protection against aphids when expressed *in planta*.

## 4. Materials and Methods

### 4.1. Antimicrobial Peptides

We used the natural scorpion AMPs UyCT1, UyCT3, UyCT5, Uy17, Uy192, and Uy234 from *U. yaschenkoi*, and Um4 from *U. manicatus* [24,26,27]. Enhanced UyCT peptides (herein named D-peptides) were modified to increase membrane affinity [28]. The UyCT group of peptides was synthesized by Biomatik Corporation (Cambridge, ON, Canada) at 98% purity. The remaining scorpion peptides were synthesized by Caslo ApS (Lyngby, Denmark) at 98% purity. All peptides were amidated at the C-terminus. The scorpion AMPs selected for this study were chosen based on their activity against human pathogenic bacteria [21,28], and those with low MICs and haemolytic values were preferred (Table 3). Three insect AMPs were tested as controls: cecropin A from the moth *Hyalophora cecropia* (Linnaeus), apidaecin from the bumblebee *Bombus pascuorum* (Scopoli), and stomoxyn from the stable fly *Stomoxys calcitrans* (Linnaeus) [37,39,40]. These insect AMPs display antibacterial activity against a broad range of Gram-negative and Gram-positive bacteria [42]. Insect AMPs were synthetized by Coring System Diagnostix (Gernsheim, Germany) at >90% purity. 

### 4.2. Aphids and the Detection of Bacterial Symbionts

*A. pisum* clone LL01 was reared on 2–3-week-old bean plants (*Vicia faba* var. minor) in a climate cabinet (KBWF 720, Binder GmbH, Tuttlingen, Germany) with a 16-h photoperiod and a day/night temperature of 24/18 °C, as previously described by [57]. Plants for experiments and aphid rearing were cultivated in a greenhouse at an average temperature of 20 °C under natural light, plus additional illumination (SONT Agro 400 W, Phillips, Eindhoven, The Netherlands) to maintain a 14-h photoperiod.

The *A. pisum* population was screened for the presence of bacterial symbionts, as previously described [58,59], with slight modifications. Total genomic DNA was isolated from individual aphids or pools of 10–20 aphids using the CTAB method [60]. Bacterial symbionts were detected by PCR using genus-specific primers to amplify 16*S* rRNA gene fragments (Table S2) [59,61]. The reaction volume was 25 μL, comprising of 4 μL DNA template (25 ng/µL), 10 µM of each primer (1 µL), 12.5 µL of GoTaq Green 2x Master Mix (Promega, Madison, WI, USA) and 6.5 µL nuclease-free water. PCR products were visualized by 1% agarose gel electrophoresis using SYBR Safe (Invitrogen, Darmstadt, Germany). Amplicons were eluted using the NucleoSpin^®^ Gel and PCR Clean-up kit (Macherey-Nagel, Düren, Germany), and sequenced for verification on a 3730xl DNA analyser (Macrogen Europe, Amsterdam, The Netherlands). Only *B. aphidicola* and *S. symbiotica* were detected in our aphid population and each individual harboured both bacterial symbionts (data not shown). The sequences were compared against NCBI databases using BLAST and deposited under accession numbers KX900450–KX900452 for *S. symbiotica* and KX910798–KX910801 for *B. aphidicola* [62].

### 4.3. Aphid Feeding with AMPs

*A. pisum* nymphs (48 h old) were fed for three days on an artificial AP3 diet in modified chambers [63,64]. The AP3 diet was mixed with the corresponding AMP or control treatment. Ten nymphs were placed in each chamber and five replicates were included per treatment. AMPs were tested at three different concentrations: 50, 250, and 500 µg/mL. Untreated aphids were fed on the control AP3 diet. Positive control treatments comprised aphids fed on the AP3 diet supplemented with the insecticide imidacloprid (5 µg/mL) or the antibiotic rifampicin (50 µg/mL) (Sigma-Aldrich, Taufkirchen, Germany) [30,64]. Imidacloprid is strongly hydrophobic, and was therefore prepared first as a highly concentrated stock (1000 µg/mL) in acetone and working solutions were diluted in the AP3 diet. The corresponding control (AP3 + acetone) was tested on the aphids, and survival was not affected when compared to AP3 diet alone or AP3 diet diluted with water (data not shown). Mortality was scored after 24, 48, and 72 h of feeding. Aphids that survived the three-day treatment were transferred to agar plates containing bean plant leaves and reared for another two weeks in order to determine the impact of the diets on survival and reproduction [65].

### 4.4. In Vitro Activity of Scorpion and Insect AMPs against Serratia Symbiotica CWBI-2.3

*S. symbiotica* strain CWBI-2.3, the only aphid symbiotic bacterium that can be cultivated in the laboratory, was purchased from the Leibniz Institute DSMZ (Braunschweig, Germany) and cultivated as recommended by the supplier. MICs were determined according to the CLSI guidelines using a broth microdilution assay in 96-well polypropylene microtiter plates. The bacteria were cultivated overnight at 28 °C using 535 medium (Tripticase soy broth) and diluted to 5 × 10^5^ CFU/mL in broth. The AMPs were dissolved in water to a concentration of 4 mg/mL and a series of two-fold dilutions was prepared in 535 broths, ranging from 500 to 4 µg/mL. *S. symbiotica* CWBI-2.3 in an unmodified medium was used as a positive control, and blanks were prepared with medium only or with medium and water (the latter to exclude any possible negative effect of water on the bacteria). The bacteria were incubated for 18 h and the absorbance at 600 nm was recorded every 20 min. The MICs were defined as the lowest concentrations of AMP causing complete bacterial growth inhibition. 

### 4.5. Relative Quantification of Bacterial Symbionts In Vivo

The density of the *B. aphidicola* and *S. symbiotica* populations in vivo was determined by qPCR. Genomic DNA was extracted from pools of five aphids, as previously described [60]. Three biological replicates were prepared per treatment. The primers used for the identification of bacterial symbionts and the reference genes are listed in Table S2 [66]. Amplifications were carried out using a StepOnePlus™ Real-Time PCR System (Applied Biosystems, Waltham, MA, USA). The reaction volume was 10 μL, comprising 2 μL of template DNA (25 ng/μL), 10 μM of each specific primer and 5 μL of SYBR Green PCR Master Mix (Applied Biosystems). Each reaction was heated to 95 °C for 10 min, followed by 40 cycles of 95 °C for 15 s and 60 °C for 60 s. Melting curve analysis was performed by increasing the temperature from 60 °C to 95 °C for 15 s, cooling to 60 °C for 60 s and heating to 95 °C for 15 s. The expression of each gene was tested in triplicate to ensure reproducibility. Relative abundance values for each symbiont were calculated by comparing the threshold cycle (Ct) of each target gene to that of the aphid ribosomal protein L32 gene [67] and efficiencies were calculated using LinReg PCR software. 

### 4.6. Localization of Bacterial Symbionts In Vivo by FISH

FISH was carried out as previously described [68], with slight modifications. Treated *A. pisum* nymphs were fixed for three days in Carnoy’s solution (6:3:1 chloroform:ethanol:glacial acetic acid) and then bleached in 6% H_2_O_2_ in 96% ethanol for 1 week. After bleaching, samples were washed in 100% ethanol and then hybridized overnight in hybridization buffer (20 mM Tris-HCl pH 8.0, 0.9 M NaCl, 0.01% sodium dodecylsulfate, 30% formamide) containing 100 nM of each fluorescent probe and 500 nM DAPI. Different probes were used to label *Buchnera* (ApisP2a) and *Serratia* (SerratiaPA) as shown in Table S2 [15]. After hybridization, samples were rinsed three times with phosphate buffered saline containing 0.3% Triton X-100 and viewed under a Leica TCS SP8 confocal microscope (Leica Microsystems, Wetzlar, Germany). We analysed a minimum of 20 samples from each treatment. The specificity of detection was confirmed using controls with no probe and specimens were pre-treated with RNase.

### 4.7. Data Analysis

All data were analysed using SPSS v17.0 software (SPSS Inc., Chicago, IL, USA) and statistical significance was defined as *p* < 0.05. For mortality assessment, we used non-parametric survival analysis (Kaplan-Meier) and multiple pairwise comparisons among different groups were carried out using log-rank tests to assess efficiency. The total number of offspring, time to reproduction, and relative numbers of bacterial symbionts were analysed using the Wilcoxon ranked sum test for non-parametric data and a paired *t*-test for parametric data. 

## Figures and Tables

**Table 1:** Effect of antimicrobial peptides (AMPs) and control treatments after three days of feeding.

Treatment		Concentration (µg/mL)	% Survival	Significance ^˄^
Insecticide	Imidacloprid	5	0	****
Antibiotic	Rifampicin	50	92.5	ns
Scorpion AMPs	UyCT1	50	96	ns
		250	76	ns
		500	28.6	****
	UyCT3	50	70	*
		250	52.5	****
		500	35	****
	UyCT5	50	68	**
		250	27.5	****
		500	10	****
	Uy17	50	75	ns
		250	4	****
		500	0	****
	Uy192	50	82	ns
		250	57.5	****
		500	12	****
	Uy234	50	85	ns
		250	0	****
		500	20	****
	Um4	50	85	ns
		250	90	ns
		500	55	****
	D3	50	72.5	*
		250	72.5	*
		500	32.5	****
	D5	50	83.7	ns
		250	42.5	****
		500	22	****
	D10	50	86	ns
		250	0	****
		500	0	****
	D11	50	86.7	ns
		250	57.1	**
		500	64	**
Insect AMPs	Apidaecin	50	100	ns
		250	90	
		500	83.3	
	Cecropin A	50	80	
		250	83.3	
		500	85	
	Stomoxyn	50	68	
		250	68	
		500	75	

^˄^ Compared to control AP3 diet (survival = 90%); ns—not significant, *p* > 0.05; * *p* ≤ 0.05; ** *p* ≤ 0.01; *** *p* ≤ 0.001; **** *p* ≤ 0.0001.

**Table 2:** Efficacy of treatments used against the bacterial symbiont *S. symbiotica* CWBI-2.3.

Compounds		MIC (In Vitro) (µg/mL)
Scorpion AMPs	UyCT1	125
	UyCT3	125
	UyCT5	125
	Uy17	250
	Uy192	500
	Uy234	>500
	Um4	>500
	D3	250
	D5	500
	D10	>500
	D11	>500
Insect AMPs	Apidaecin	>500
	Cecropin A	>500
	Stomoxyn	>500
Antibiotics	Rifampicin	50

MIC—minimal inhibitory concentration.

**Table 3:** List of AMPs and control compounds tested against *A. pisum* and its bacterial symbionts.

Compounds		Sequence or Chemical Formula
Scorpion AMPs	UyCT1	GFWGKLWEGVKNAI
	UyCT3	ILSAIWSGIKSLF
	UyCT5	IWSAIWSGIKGLL
	Uy17	ILSAIWSGIKGLL
	Uy192	FLSTIWNGIKGLL
	Uy234	FPFLLSLIPSAISAIKRL
	Um4	FFSALLSGIKSLF
	D3	LWGKLWEGVKSLI
	D5	GFWGKLLEGVKKAI
	D10	FPFLKLSLKIPKSAIKSAIKRL
	D11	GFWGKLWEGVKNAIKKK
Insect AMPs	Apidaecin	GNRPVYIPPPRPPHPRL
	Cecropin A	KWKL FKKIEKVGQN IRDGIIKAGPAVAVVGQATQIA
	Stomoxyn	RGFRKH FNKLVKKVKH TISETAHVAKDTAVIAGSGA AVVAAT
Antibiotic	Rifampicin	C_43_H_58_N_4_O_12_ (CAS number 13292-46-1)
Insecticide	Imidacloprid	C_9_H_10_ClN_5_O_2_ (CAS number 138261-41-3)

## Supplementary Materials

The following are available online at www.mdpi.com/2072-6651/9/9/261/s1, Figure S1: Scheme representing the methodology followed; Table S1: Statistical data for Kaplan-Meier analysis shown in Figure 1; Table S2: List of primers and probes.
