# Supplementary material for: Orally Delivered Scorpion Antimicrobial Peptides Exhibit Activity against Pea Aphid (Acyrthosiphon pisum) and Its Bacterial Symbionts
Source: Toxins (Basel). 2017 Aug 24;9(9):261. doi: 10.3390/toxins9090261 (PMC5618194; doi:10.3390/toxins9090261)
Supplement: Supplementary file 1 [file toxins-09-00261-s001.pdf]

Supplementary Materials: Orally Delivered Scorpion Antimicrobial Peptides Exhibit Activity against Pea Aphid (*Acyrthosiphon pisum*) and Its Bacterial Symbionts

Karen Luna-Ramirez, Marisa Skaljac, Jens Grotmann, Phillipp Kirfel and Andreas Vilcinskas

Table S1. Statistical data for Kaplan-Meier analysis shown in Figure 1.

Treatment	Mean[a]			
	Estimate	Stdandard error	95% Confidence Interval	
			Lower Bound	Upper Bound
Control	2.880	0.054	2.774	2.986
Imidacloprid (5 µg/mL)	1.960	0.070	1.823	2.097
Rifampicin (50 µg/mL)	2.925	0.042	2.843	3.007
Cecropin A (500 µg/mL)	2.925	0.046	2.836	3.014
Apidaecin (500 µg/mL)	2.967	0.037	2.895	3.038
Stomoxyn (500 µg/mL)	2.904	0.043	2.820	2.987
UyCT5 (500 µg/mL)	2.180	0.084	2.015	2.345
UyCT1 (500 µg/mL)	2.653	0.086	2.485	2.821
UyCT3 (500 µg/mL)	2.125	0.136	1.859	2.391
Um4 (500 µg/mL)	2.475	0.131	2.218	2.732
Uy17 (500 µg/mL)	2.525	0.080	2.368	2.682
Uy192 (500 µg/mL)	2.380	0.069	2.244	2.516
Uy234 (500 µg/mL)	2.600	0.081	2.441	2.759
D3 (500 µg/mL)	2.525	0.108	2.313	2.737
D5 (500 µg/mL)	2.680	0.073	2.537	2.823
D10 (500 µg/mL)	1.979	0.094	1.795	2.162
D11 (500 µg/mL)	2.720	0.077	2.569	2.871

a. Estimation is limited to the largest survival time if it is censored.

Table S2. List of primers and probes.

Method used	Target gene	Primer/probe name	Primer/probe sequence (5'→3')	Annealing temp (°C)/ Product size (bp) E /R^2 (when applicable)	Reference
PCR	*Buchnera* (16*S* rRNA)	Buch16S1F Buch16S1R	GAGCTTGCTCTCTTTGTCGGCAA CTTCTGCGGGTAACGTCACGAA	66/430	Tsuchida et al., 2002 [1]
	Serratia (16*S* rRNA)	16SA1 PASScmp	AGAGTTTGATCMTGGCTCAG GCAATGTCTTATTAACACAT	59/480	Fukatsu & Nikoh, 1998 [2]
qPCR	Ribosomal protein L32	rpl32_F rpL32_R	AGTATCGCCCAACAATTATCA CTTGAATCGTCTTCGGACT	60/131 E=107.19% R^2= 0.998	Sapountzis et al., 2014 [3]
	Buchnera (16*S* rRNA)	Buchq1F Buchq1R	AGCGTGGGGAGCAAACAGGA ACATGCTCCACCGCTTGTGC	60/185 E=94.15% R^2=0.998	This study
	Serratia (16*S* rRNA)	Serq1_146F Serq1_269 R	AGCGTGGGGAGCAAACAGGA ACTCCCCAGGCGGTCGATTT	60/123 E=93.14% R^2=0.999	This study
FISH	*Buchnera* (16*S* rRNA)	ApisP2a - cy5	CCTCTTTTGGGTAGATCC		Koga et al., 2003 [4]
	Serratia (16*S* rRNA)	SerratiaPA -cy3	GACATCGTTTACAGCGTGGA		This study

E= quantivative PCR efficiency, R^2= coefficient of determination

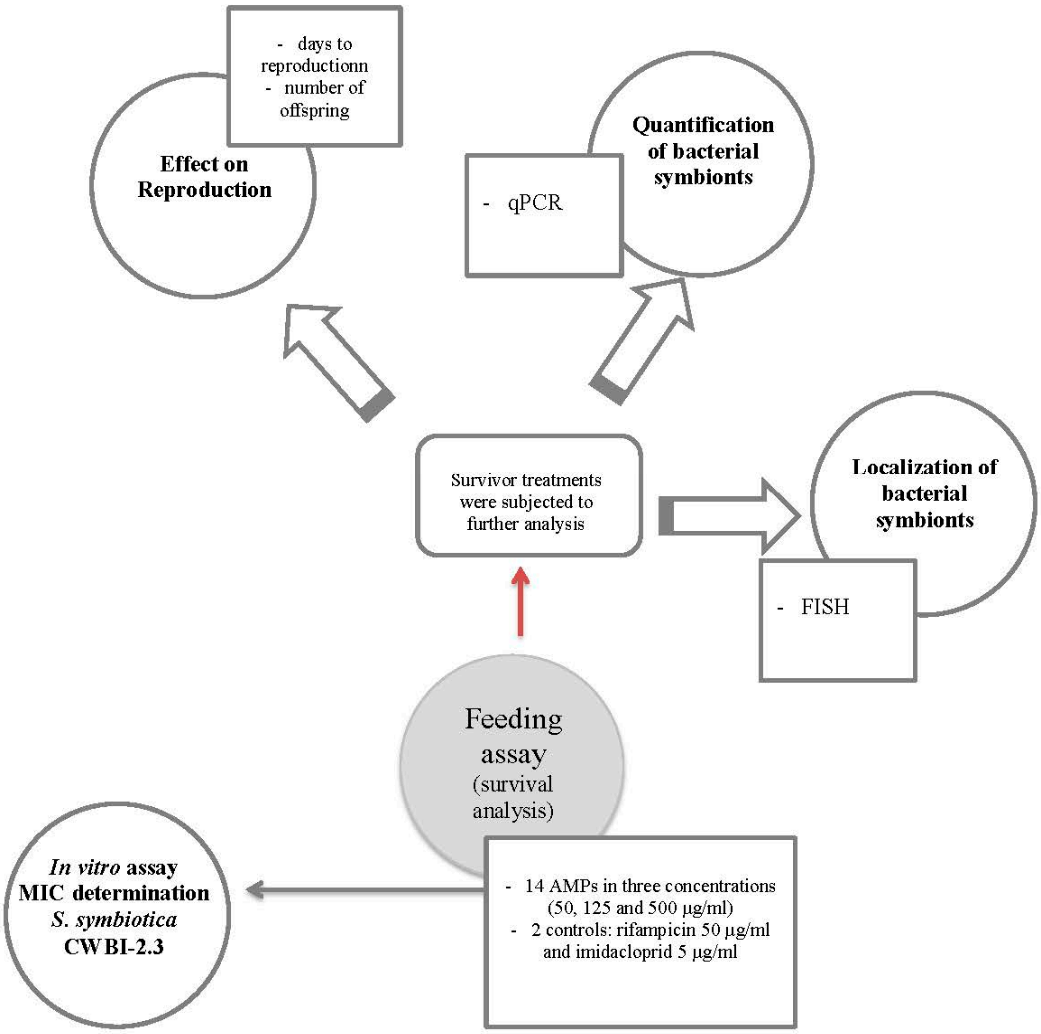

Figure S1. Scheme representing the methodology followed.

References

1. Tsuchida, T.; Koga, R.; Shibao, H.; Matsumoto, T.; Fukatsu, T. Diversity and geographic distribution of secondary endosymbiotic bacteria in natural populations of the pea aphid, *Acyrthosiphon pisum*. *Mol. Ecol.* **2002**, *11*, 2123–2135.
2. Fukatsu, T.; Nikoh, N. Two intracellular symbiotic bacteria from the mulberry psyllid anomoneura mori (insecta, homoptera). *Appl. Environ. Microbiol.* **1998**, *64*, 3599–3606.
3. Sapountzis, P.; Duport, G.; Balmand, S.; Gaget, K.; Jaubert-Possamai, S.; Febvay, G.; Charles, H.; Rahbe, Y.; Colella, S.; Calevro, F. New insight into the RNA interference response against cathepsin-l gene in the pea aphid, *Acyrthosiphon pisum*: Molting or gut phenotypes specifically induced by injection or feeding treatments. *Insect Biochem. Mol. Biol.* **2014**, *51*, 20–32.
4. Koga, R.; Tsuchida, T.; Fukatsu, T. Changing partners in an obligate symbiosis: A facultative endosymbiont can compensate for loss of the essential endosymbiont *Buchnera* in an aphid. *Proc. Biol. Sci.* **2003**, *270*, 2543–2550.
